# Supplementary material for: Red LED Light Irradiation Increases the Resistance Against Environmental Stress of Frozen Bovine Sperm Thawed in Suboptimal Conditions
Source: Animals (Basel). 2025 Nov 20;15(22):3353. doi: 10.3390/ani15223353 (PMC12649307; doi:10.3390/ani15223353)
Supplement: Supplementary file 1 [file animals-15-03353-s001.zip › animals-3945433-supplementary.pdf]

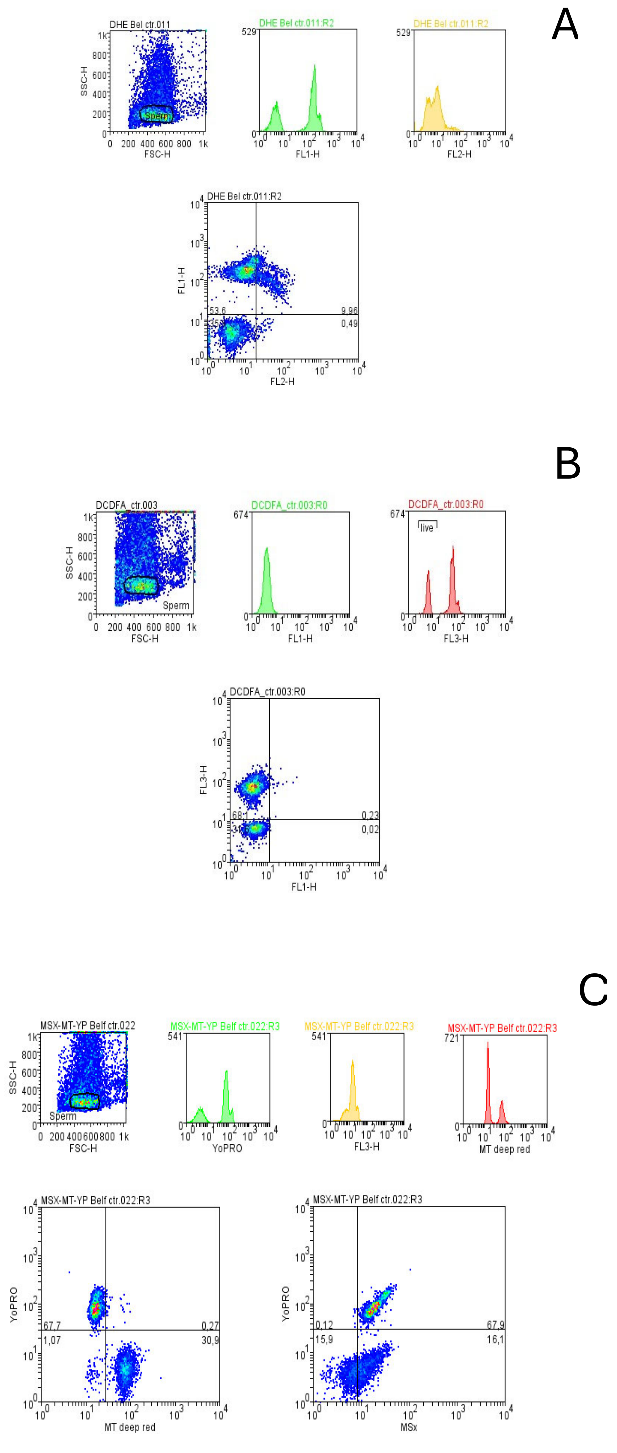

Suppl. Fig. S1. Representative plots for flux cytometer results regarding analyses of DHE/YO-PRO-1, CM-H2DCFDA/PI, and MitoSOX/Mitotracker deep red/YO-PRO-1 data. Figure shows representative plots obtained from CONTROL thawed samples. A: DHE/Yo-PRO-1 plots. B: CM-H2DCFDA/PI plots. C: MitoSOX/Mitotracker deep red/YO-PRO-1 plots. Showed plots are representative for all 16 experiments, including samples from both PHOTO and ET groups.
